# MMP-1 expression has an independent prognostic value in breast cancer

**DOI:** 10.1186/1471-2407-11-348

**Published:** 2011-08-11

**Authors:** Pia Boström, Mirva Söderström, Tero Vahlberg, Karl-Ove Söderström, Peter J Roberts, Olli Carpén, Pirkko Hirsimäki

**Affiliations:** 1 University of Turku and Turku University Central Hospital, Department of Pathology, Kiinamyllynkatu 10, 20520 Turku, Finland; 2 University of Turku, Department of Biostatistics, Lemminkäisenkatu 1, 20014 Turku, Finland; 3 Turku University Central Hospital, Department of Surgery, Kiinamyllynkatu 4 - 8, 20520 Turku, Finland

## Abstract

**Background:**

Breast cancer consists of a variety of tumours, which differ by their morphological features, molecular characteristics and outcome. Well-known prognostic factors, e.g. tumour grade and size, Ki-67, hormone receptor status, HER2 expression, lymph node status and patient age have been traditionally related to prognosis. Although the conventional prognostic markers are reliable in general, better markers to predict the outcome of an individual tumour are needed.

Matrix metalloproteinase-1 (MMP-1) expression has been reported to inversely correlate with survival in advanced cancers. In breast cancer MMP-1 is often upregulated, especially in basal-type breast tumours. The purpose of this retrospective study was to analyse MMP-1 expression in breast cancer cells and in cancer associated stromal cells and to correlate the results with traditional prognostic factors including p53 and bcl-2, as well as to patient survival in breast cancer subtypes.

**Methods:**

Immunohistochemical analysis of MMP-1, ER, PR, Ki-67, HER2, bcl-2, p53 and CK5/6 expression was performed on 125 breast cancers. Statistical analyses were carried out using Kruskal-Wallis and Mann-Whitney -tests. In pairwise comparison Bonferroni-adjustment was applied. Correlations were calculated using Spearman rank-order correlation coefficients. Kaplan-Meier survival analyses were carried out to compare breast cancer-specific survival curves. Factors significantly associated with disease-specific survival in univariate models were included in multivariate stepwise.

**Results:**

Positive correlations were found between tumour grade and MMP-1 expression in tumour cells and in stromal cells. P53 positivity significantly correlated with MMP-1 expression in tumour cells, whereas HER2 expression correlated with MMP-1 both in tumour cells and stromal cells. MMP-1 expression in stromal cells showed a significant association with luminal A and luminal B, HER2 overexpressing and triple-negative breast cancer subtypes.

**Conclusions:**

The most important finding of this study was the independent prognostic value of MMP-1 as well as Ki-67 and bcl-2 expression in tumour cells. Our study showed also that both tumoural and stromal MMP-1 expression is associated with breast tumour progression and poor prognosis. A significant difference of MMP-1 expression by cancer associated stromal cells in luminal A, luminal B and triple-negative breast cancer classes was also demonstrated.

**Please see related commentary article **http://www.biomedcentral.com/1741-7015/9/95

## Background

Breast cancer consists of a variety of tumour types, which differ by their morphology, molecular characteristics and clinical outcome. It is the leading cause of cancer death among women aged 20-59 years in high-income countries [1]. The prognostic factors that indicate disease outcome include tumour grade and size, proliferation index Ki-67, hormone receptor status, HER2 expression, lymph node status and patient age. In addition, several markers including p53 [2] and bcl-2 [3,4] expression have been shown to associate with survival.

The current breast cancer classification is based on morphological features [5]. Newer approaches to molecular classification by gene expression profiling have identified five distinct subclasses [6,7]. Two of them are ER positive (luminal A and B) and three ER negative (HER2 overexpression, normal breast-like and basal-like types) with different prognoses and treatment responses to current therapies. Basal-like cancers are negative for hormone receptors, but positive for basal cytokeratins. Triple-negative breast cancers lack expression of HER2, estrogen and progesterone receptors. The majority of triple-negative breast cancers carry the basal-like molecular profile [8].

Although well-documented classical prognostic markers are reliable in general, better markers to predict the outcome of an individual tumour are needed. It would be especially important to identify patients with favourable outcome and to save them from treatment side-effects. On the other hand, tumours that have capability to metastasize need targeted treatment and intensified follow-up.

Tumour invasion and metastasis is a multistage process, which includes tumour growth, local proteolysis and migration of the tumour cells through the degraded tissue [9]. All these steps involve interaction between tumour cells and the extracellular matrix (ECM). Local proteolysis is carried out by proteinases produced by the tumour cells or by the surrounding stromal cells. Matrix metalloproteinases (MMPs) are a family of enzymes consisting of 28 members [10] capable of degrading essentially all macromolecules of the ECM [11]. The activity of MMPs is controlled extracellularly by tissue inhibitors of matrix metalloproteinases (TIMPs) [12]. A number of studies have demonstrated a correlation between MMP expression and the invasive potential of human cancer [13]. Furthermore, the ratio of MMPs to TIMPs has been related to the prognosis of several human tumours, including breast cancer [14,15]. Recent studies have also shown that MMPs' functional genetic polymorphisms may contribute to breast cancer risk [16]. In most malignant tumours, stromal fibroblasts have been shown to be the predominant source of MMPs [17], but there is also evidence that MMPs are produced by cancer cells [18].

MMP-1 has been described in a wide range of advanced cancers with a significant negative correlation with survival [18,19]. MMP-1 is often upregulated in breast cancer, especially in basal-type tumours [20], and is supposed to be critically involved in metastatic dissemination [21,22]. Recent reports suggest that MMP-1 associates with a shortened relapse free survival [23] and poor outcome in breast cancer [20].

In most previous studies MMP-1 expression has been analyzed at mRNA rather than at protein level. Therefore, the purpose of this retrospective study was to analyse the exact location of MMP-1 expression both in breast cancer cells as well as in cancer associated stromal cells and to correlate the results with traditional prognostics factors and with bcl-2 and p53 expression in different breast cancer subtypes. In addition, we wanted to correlate MMP-1 expression to breast cancer-specific survival during an extensive follow-up time.

## Methods

### Patients and tissue material

The material consisted of 118 female breast cancer patients whose tissue samples were available (mean age at surgery was 57,5 years, range 30-90 years) and who were operated and treated at Turku University Central Hospital during 1985-1994. In addition, to increase the number of basal-like subtype seven patients operated in 2007 were included in the study. Due to the retrospective nature of the study, informed consents could not be obtained from individual patients. The permission to use the tissue material without informed content was given by the local and national regulatory authorities, Turku University Hospital Ethics committee (no 241/2005) and the Finnish National Authority for Medicolegal Affairs (no 4424/32/300/02). Follow-up information on life status was collected for each case (Central Statistical Office of Finland). Tumour characteristics including tumour size, grade and lymph node status were obtained from the pathology database (Table 1). None of the patients received radiation or chemotherapy before operation. All breast tumours were invasive carcinomas and most of them were symptomatic.

**Table 1:** Patients and tumour characteristics

Variable	Number of patients (%)
**Number of the patients**	125 (aged 30-90, mean 57, 5)
**Grade**	
I	10 (8%)
II	66 (52, 8%)
III	49 (39, 2%)
**Axillary nodal status**	
N0	64 (51, 2%)
≥N1	50 (40%)
Unknown	11 (8, 8%)
**Estrogen receptor status (ER)**^1)^	
Positive	80 (64%)
Negative	45 (36%)
**Progesterone receptor status (PR)**^1)^	
Positive	82 (65, 6%)
Negative	43 (34, 4%)
**Ki-67 status**^2)^	
low ≤ 15%	63 (50,4%)
intermediate 16-30%	41 (32,8%)
high > 30%	20 (16%)
one value missing	1 (8%)
**Histologic type**	
Ductal	110 (88%)
Lobular	10 (8%)
Subtypes	5 (4%)
**Her2**^3)^	
IHC positive (2+ and 3+)	25 (20%)
IHC negative (0 and 1+)	100 (80%)
**CK 5/6**^4)^	
Triple-negative (ER-, PR-, Her2-)	35 (28%)
Basal-like carcinoma (ER-, PR-, Her2-, CK5/6+)	20 (16%)
**Treatment after operation**	
Chemotherapy 27	(21,6%)
Radiation 59	(47,2%)
Hormonal therapy 24	(19,2%)

^1,4) ^Cut off point used for ER and PR immunohistochemistry is 10% of positively stained tumour nuclei and 10% cytoplasmic staining for CK5/6.

^2) ^Proliferation index according to St Gallen Consesus (Goldhirsch et al 2009).

^3) ^Scoring of HER2 immunohistochemistry: Score 0: no staining is observed or cell membrane staining is observed in less than 10% of tumour cells. Score 1+: a faint perceptible membrane staining can be detected in more than 10% of the tumour cells or cells are only stained in part of their membrane. Score 2+: a weak to moderate complete membrane staining is observed in more than 10% of the tumour cells. Score 3+: a strong complete membrane staining is observed in more than 10% of the tumour cells.

Four μm thick serial sections from formalin-fixed, paraffin-embedded tumour tissue were cut and stained with haematoxylin and eosin. Slides were reviewed to confirm the diagnosis. The tumour histology was assessed according to the WHO classification [5] and tumour grading (I-III) was based on the recommendations made by Elston and Ellis 1991 [24].

### Immunohistochemistry

Immunohistochemical staining of MMP-1, ER, PR, Ki-67, HER2, bcl-2, p53 and CK5/6 was carried out with TechMate 500+ immunostainer using monoclonal antibodies and a peroxidase/diaminobenzidine LSAB+ or EnVision detection kit (DAKO). Immunodetection of MMP-1 was performed using mouse monoclonal MMP-1 antibody (Onkogene) with dilution 1:100 and 10 mM TRIS-HCl with 1 mM EDTA (pH9) as a pretreatment. Immunohistochemical staining of ER, PR, Ki-67, HER2, bcl-2, p53 and CK5/6 were performed as described [25]. The used MMP-1 antibody recognises both the latent and the active forms of MMP-1 protein.

**Figure 1:**
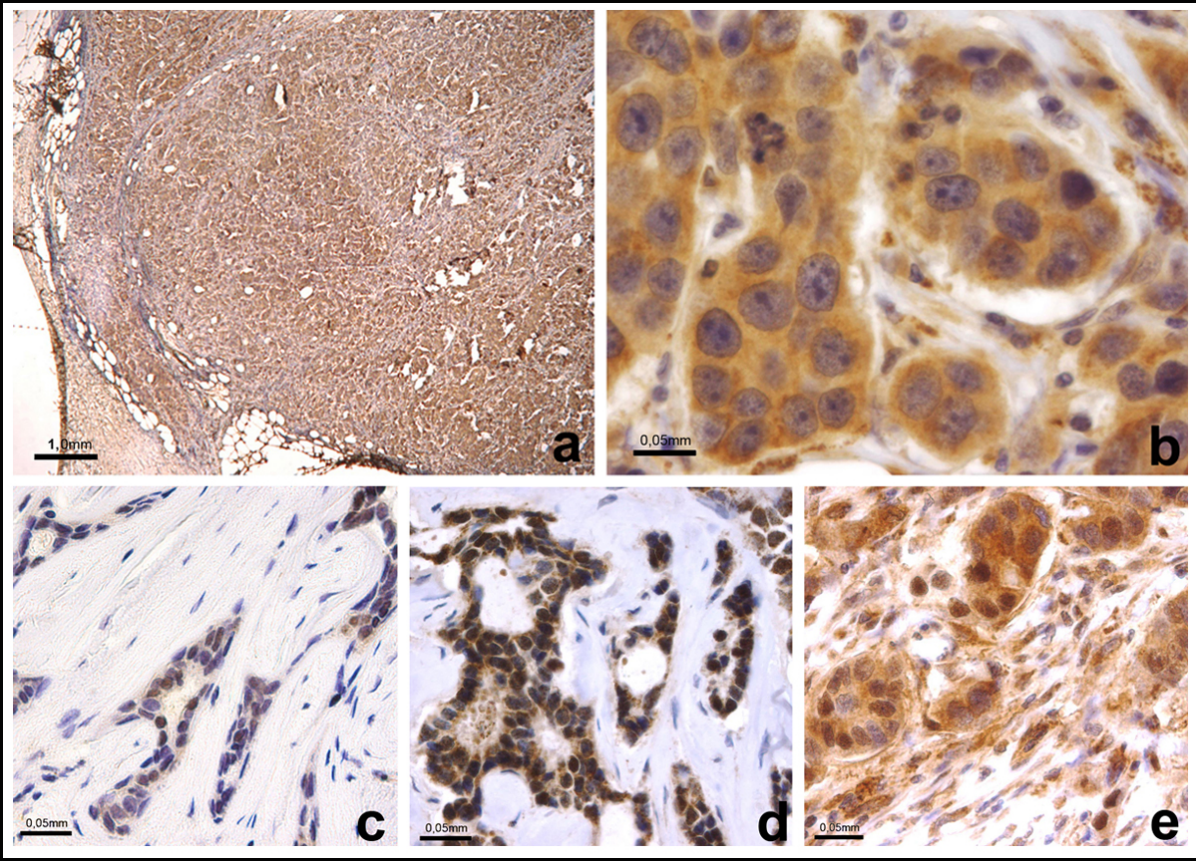
**Immunohistochemical staining of MMP-1 in breast cancer**. a-b) Immunohistochemical staining of MMP-1 in infiltrating ductal carcinoma GIII. The staining is observed both in nuclei and in the cytoplasm. c) Breast carcinoma cells with positive MMP-1 nuclear staining and with d) positive nuclear and cytoplasm staining and with e) positive tumour cells and cancer associated stromal cells.

MMP-1 (Figures 1a-e), p53, HER2, ER, PR, Ki-67 and bcl-2 staining was evaluated from cells at the border of the most cellular part of the tumour. Areas showing necrosis or inflammation were excluded from the analysis. For MMP-1 expression the number of immunopositive tumour cells per 100 malignant cells (0-100%) from three separate cell rich areas were calculated with a 40 × objective independently by two observers (PB, PH) without any knowledge of the clinical data and the mean value of immunopositive areas was recorded. In addition to tumour cells MMP-1 staining in non-lymphoid stromal cells was evaluated in the same way. The percentage of tumour cells with nuclear staining for hormonal receptors, Ki-67 and p53 was also recorded. For bcl-2, cytoplasmic staining was scored and the percentage of positive tumour cells was recorded. HER2 expression was evaluated as membrane staining of invasive tumour cells and scored to four classes (0/1+/2+/3+). Triple-negative cases (ER-, PR- and HER2-) were stained with basal cytokeratin CK5/6, and expression was considered as positive, if at least 10% of the cancer cells showed cytoplasmic and/or membranous staining.

### Statistical analyses

Statistical analyses were carried out using Kruskal-Wallis and Mann-Whitney -tests. In pairwise comparison Bonferroni-adjustment was applied. Correlations were calculated using Spearman rank-order correlation coefficients. Non-parametric test was applied because of non-normal distribution of responses.

Breast cancer-specific survival time was determined from time of diagnosis until death from breast cancer. Patients who were alive at the end of the follow-up on October 2009 or died of other causes were used as censored values in survival analyses. Kaplan-Meier survival analyses were carried out to compare breast cancer-specific survival curves. Univariate Cox regression model was used to examine prognostic factors for breast cancer-specific survival. Factors significantly associated with disease-specific survival in univariate models were included in multivariate stepwise (inclusion and exclusion criteria p = 0.05) Cox regression model. Results are expressed using hazard ratios (HR) with 95% confidence intervals (CI) and P-values less than 0.05 were considered as significant. The statistical analyses were carried out using SAS/STAT(r) software, Version 9.1.3 SP4 of the SAS System for Windows.

Cox univariate method was used to calculate breast cancer-specific survival analysis for 118 patients. The seven patients from this century were not included in survival data, because their treatment might have been different from earlier decades and because of the short follow-up. To examine interactions of different prognostic factors in a multivariate analysis, we used the Cox regression model. Parameters that achieved statistical significance for disease-specific survival in the log-rank test were included in the multivariate analysis.

## Results

### Immunohistochemical staining and statistical analyses

**Figure 2:**
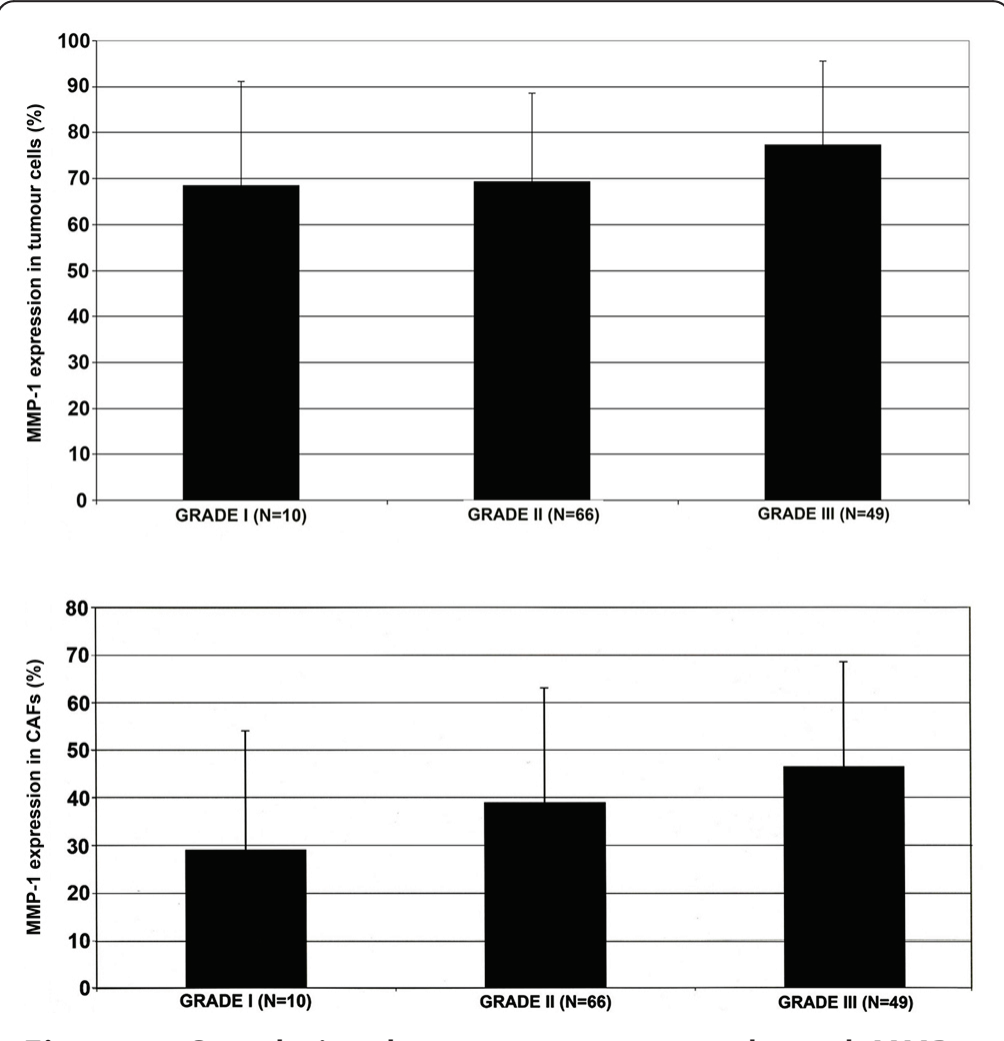
**Correlation between tumour grade and MMP-1 immunoreactivity**. The histograms present an association between the histological grade of the breast cancer and the immunohistochemical expression of MMP-1. P-values for MMP-1 expression in tumour cells: overall p = 0.023, grade I vs. II p = 1.0, grade I vs. III p = 0.81, grade II vs. III p = 0.0177. P-values for MMP-1 expression in stromal cells: overall p = 0.0493, grade I vs. II p = 0.4839, grade I vs. III p = 0.1008, grade II vs. III p = 0.2637.

MMP-1 positivity in tumour cells and in stromal cells was observed in all analyzed tumours. The MMP-1 expression in tumour cells ranged from 10% to 95% and in stromal cells from 5% to 80%. In tumour cells the MMP-1 expression was positive in 7.2% with value ≤ 30%, in 8% with value ≤ 50%, in 31.2% with value ≤ 70% and in 53.6% with value over 70%. The MMP-1 positivity was lower in stromal cells, where 47.6% of cases were positive with value ≤ 30%, 14.5% positive with value ≤ 50%, 30.6% positive with value ≤ 70% and 7.3% positive with value over 70%. A positive correlation was seen between tumour grade and MMP-1 expression in tumour cells (r = 0.23, p = 0.0101, Figure 2) and in stromal cells (r = 0.21, p = 0.0170, Figure 2), the higher grade tumours showing stronger MMP-1 positivity.

The association between MMP-1 expression and traditional prognostic factors was evaluated. HER2 immunoreactivity correlated with MMP-1 positivity both in stromal cells (r = 0.25, p = 0.0050) and tumour cells (r = 0.22, p = 0.0121). Using a dicotomised HER2 grouping into immunonegative (0 - 1+ classes) and immunopositive (2+ - 3+ classes) groups, we also found a significant positive association between HER2 and MMP-1 expression in tumour cells (p = 0.0086) and in stromal cells (p = 0.0038). MMP-1 positivity in cancer cells or in stromal cells did not show significant correlation with estrogen or progesterone receptors. P53 had a significant positive correlation with MMP-1 expression in tumour cells (r = 0.23, p = 0.0113), but not in stromal cells. Ki-67 or Bcl-2 did not show any significant correlation with MMP-1 staining in tumour cells or in stromal cells.

**Figure 3:**
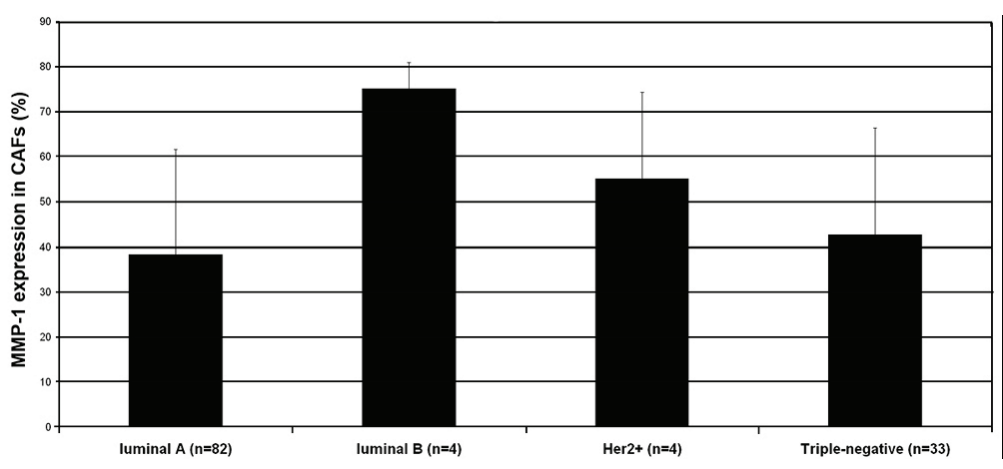
**MMP-1 immunoreactivity of stromal cells of various breast cancer subtypes**. Immunohistochemically detected expression of MMP-1 in stromal cells with significant differences in breast cancer subtypes. P-values for overall difference p = 0.0129, luminal A vs. luminal B p = 0.0258, luminal A vs. Her2 p = 0.8334, luminal A vs. triple-negative p = 1.00, luminal B vs. Her2 p = 0.3792, luminal B vs. triple-negative p = 0.0336.

We further evaluated the association between MMP-1 expression and the different molecular breast cancer subtypes. MMP-1 positivity in stromal cells showed significant differences (p = 0.0129) between breast cancer subtypes. In luminal B subtype, the MMP-1 expression in stromal cells was higher than in luminal A subtype (p = 0.0258), and also the luminal B subtype stromal cells showed higher MMP-1 positivity than triple-negative breast cancer cells (p = 0.0336) (Figure 3). MMP-1 expression in cancer cells did not show any association with these breast cancer subtypes. No association with MMP-1 positivity in tumour cells or in stromal cells was found when tumours were divided to triple-negative and non-triple-negative groups. Nor was any association found, when basal-like and non-basal-like groups and MMP-1 expression in tumour cells or in stromal cells were analyzed.

### Survival analysis

The median follow-up period of the survived patients was 20 years (range, from 17 to 24 years). From the patients included in the survival analyses 44 patients (37%) were still alive, 51 (43%) had died from breast cancer and 23 (19%) from some other cause during the follow up time.

**Figure 4:**
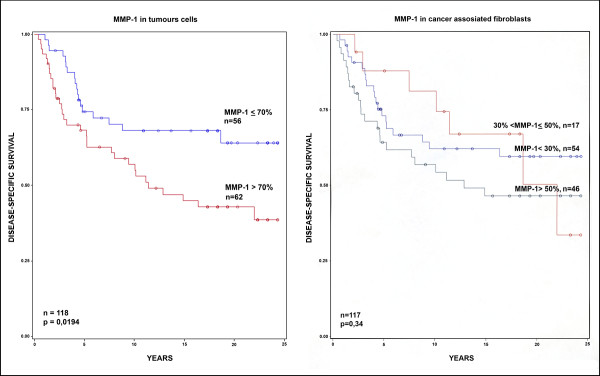
**Influence of MMP-1 expression in tumour cells and in stromal cells on survival among women with breast cancer**.

There was a statistically significant difference in breast cancer-specific survival between tumours with high vs. low expression of MMP-1 in tumour cells (cut-off value 70%, p = 0.0171) (Figure 4), tumour grade I vs. III (p = 0.0099), triple-negative vs. non-triple-negative breast cancers (p = 0.0137), basal-like vs. non-basal-like breast cancers (p = 0.0103), low vs. high bcl-2 expression (cut-off value 25%, p = 0.0003) and high vs. low estrogen receptor expression (cut-off value 10%, p = 0.0013). The difference in breast cancer-specific survival between Ki-67 subcategories (≤15% and > 15%) were also statistically significant (p = 0.0022). There was no statistically significant difference in breast cancer-specific survival between MMP-1 positivity in stromal cells with any cut-off value (Figure 4). Neither did patient age or p53 expression associate with breast cancer-specific survival.

The potential independent prognostic value of MMP-1 protein expression in tumour cells was evaluated by stepwise Cox regression analysis. Ki-67 expression and high MMP-1 positivity in tumour cells (cut-off 70%) were significantly associated with poor breast cancer-specific survival (Table 2). Positive bcl-2 status was a significant positive prognostic factor for breast cancer-specific survival (Table 2).

**Table 2:** Disease specific- survival analysis at 24 years follow-up

Variable	n	HR (95%CI)	p (univariate)	HR (95%CI)	p (multivariate)
**ER > 10%**	118	0.41 (0.24-0.72)	0.0018*		
**PR > 10%**	118	0.71 (0.40-1.25)	0.2322		
**Ki-67 > 15%**	117	2.35 (1.34-4.13)	0.0030*	2.01 (1.12-3.59)	0.0186*
**Bcl-2 > 25%**	118	0.33 (0.18-0.63)	0.0007*	0.45 (0.23-0.86)	0.0158*
**Triple-neg**	118	2.05 (1.15-3.68)	0.0158*		
**Basal-type**	118	2.34 (1.20-4.58)	0.0127*		
**p53 > 10%**	118	1.73 (0.98-3.06)	0.0601		
**MMP-1 tumour cells > 70%**	118	1.99 (1.12-3.53)	0.0194*	1.81 (1.01-3.22)	0.0438*
**MMP-1 fibroblasts > 30%**	118	1.35 (0.77-2.39)	0.2948		

HR = Hazard Ratio

P-value less than 0.05 (*) were considered as significant.

Tumour grade was not included in disease specific -survival analysis, because none of grade I patients died of breast cancer.

## Discussion

Invasion of tumour cells is a multistep process that involves proteolytic activity and migration of tumours cells through the degraded ECM. The expression of MMP-1 is characteristic for many types of malignant tumours, including breast cancer. Upregulation of MMP-1 was recently indicated to associate with poor outcome in breast cancer [20]. In this study, we evaluated MMP-1 expression by immunohistochemistry using original tissue sections instead of tissue microarrays, and correlated it with different breast cancer subtypes and with traditional prognostics factors and patient survival.

Our results demonstrate MMP-1 positivity both in the nuclei and in the cytoplasm of breast cancer cells and in the stromal cells, often termed as cancer-associated fibroblasts (CAFs). Our results showed a higher MMP-1 expression in tumour cells than in stromal cells. Earlier studies have demonstrated elevated nuclear MMP-1 expression in tumour cells [26], as well as in stromal fibroblasts [15,23,27]. Yet the results are conflicting and can be explained by the use of different tissue handling, fixation and incubation conditions as well as different antibodies used [28]. Also the scoring criteria for each antibody should be standardized. The antibody we used recognises both the latent and active forms of MMP-1 protein.

In our study, MMP-1 expression in tumour cells and in stromal cells showed a significant association with tumour grade. This finding is partially in line with previous data [29], where CAFs showed a correlation with tumour stage. Earlier studies have also shown elevation of MMP-1 mRNA in high-grade tumours when compared to low-grade tumours [20,30], but also opposite results on MMP-1 immunohistochemical expression and tumour grade exist [28].

In previous studies high expression on MMP-1 by stromal cells correlated with the occurrence of metastasis [23] suggesting that tumoural stromal tissue is important in cancer progression [31]. In our study, the expression of MMP-1 both in tumour cells and in stromal cells and especially the correlation between the expression and tumour grade may indicate an impact of MMP-1 on cellular properties such as growth, death, and migration [32]. MMPs are known to cleave not only structural components of the ECM, but also growth factor precursors, cell surface receptors, cytokines and cell adhesion molecules [18,33].

Hormone receptors are associated with a favourable prognosis and predict response to endocrine treatment [34]. In our analysis the MMP-1 positivity did not show any correlation with hormone receptors. However, we found a negative, but not a significant correlation between tumour cell MMP-1 expression and ER expression. MMP-1 expression has previously been reported to be significantly increased in ER-negative breast cancer [20,35]. In a previous study MMP-1 expression and PR expression had a statistically significant inverse correlation with CAFs and cancer cells [29]. Also the positive correlation between HER2 and MMP-1 expression both in tumour cells and stromal cells in our study shows that MMP-1 is associated with poor outcome in breast cancer. Our result showing the correlation between p53 and MMP-1 expression in tumour cells is in accordance with tumour aggressiveness.

One important finding of the present study is the significantly different MMP-1 expression in stromal cells of different breast cancer subtypes (Figure 3). In our study MMP-1 overexpression in tumour cells did not have any significant differences within breast cancer subtypes. On the other hand, we did not find any association between luminal or basal-like breast tumours and MMP-1 positivity in tumour cells. Similar results have been presented by González [36]. However, MMP-1 mRNA is shown to be up-regulated in basal-type breast cancers when compared with non-basal-type breast cancers [20].

Few studies have correlated MMP-1 expression with breast cancer-specific survival. In our study the follow up -time for breast cancer-specific survival was extensively long being over 20 years. We found that MMP-1 positivity in tumour cells with a 70% cut-off level had a significant association with survival in univariate analyses (Figure 4). The breast cancer-specific survival curve continued to decrease even during the extended follow-up period. In addition, tumour grade, triple-negative and basal-like breast cancers, ER, Ki-67 and bcl-2, were associated with breast cancer-specific survival in our study. In a multivariate analysis the presence of MMP-1 positivity in tumour cells had an independent prognostic value as did Ki-67 and bcl-2 immunoreactivity. No statistically significant difference was found in breast cancer-specific survival in the expression of MMP-1 in stromal cells with any cut-off values (Figure 4).

## Conclusions

In summary, our study shows that both tumoural and stromal MMP-1 positivity is associated with breast tumour progression and poor prognosis. Our findings show a significant difference in MMP-1 positivity of cancer associated stromal cells between luminal A, luminal B and triple-negative breast cancer types. The most important finding of the present work is that the MMP-1 expression of tumour cells carries an independent prognostic value in breast cancers. These results suggest that MMP-1 expression of both stromal and tumour cells may control breast cancer progression. Further investigation with larger patient cohorts is needed to better understand the function of MMP-1 in tumour cells.

## List of abbreviations

CAF: cancer associated fibroblast; DC: ductal carcinoma; LC: lobular carcinoma; ECM: extracellular matrix; ER: estrogen receptor; HER2: human epidermal growth factor receptor 2; PR: progesterone receptor; CK5/6: cytokeratin 5/6; MMPs: matrix metalloproteinases; TIMPs: tissue inhibitors of matrix metalloproteinases

## Competing interests

The authors declare that they have no competing interests.

## Authors' contributions

PB participated in the design of the study, carried out the screening of the tumour material and the evaluation of the immunohistochemical staining and drafted the manuscript. MS participated in the study design and helped to draft the manuscript. TV coordinated and performed the statistical analysis and helped to draft the manuscript. KOS and PJR participated in the design of the study. PJR and OC helped to draft the manuscript. PH participated in the design of the study, carried out the evaluation and validation of the immunohistochemical staining and helped to draft the manuscript. All authors have read and approved the final manuscript.

## Pre-publication history

The pre-publication history for this paper can be accessed here:

http://www.biomedcentral.com/1471-2407/11/348/prepub

## References

[B1] WHO Women's health 2009 Fact sheet No 334

[B2] Malamou-Mitsi V Gogas H Dafni U Bourli A Fillipides T Sotiropoulou M Vlachodimitropoulos D Papadopoulos S Tzaida O Kafiri G Kyriakou V Markaki S Papaspyrou I Karagianni E Pavlakis K Toliou T Scopa CD Papakostas P Bafaloukos D Christodoulou C Fountzilas G Evaluation of the prognostic and predictive value of p53 and Bcl-2 in breast cancer patients participating in a randomized study with dose-dense sequential adjuvant chemotherapy Ann Oncol 2006 17 1504 1511 10.1093/annonc/mdl147 16968874

[B3] Joensuu H Pylkkänen L Toikkanen S Bcl-2 Protein Expression and Long-Term Survival in Breast Cancer Am J Pathol 1994 145 1191 1198 7977649 PMC1887415

[B4] Martinez-Arribas F Alvarez T Del Val G Martin-Garabato E Nunez-Villar MJ Lucas R Sanchez J Tejerina A Schneider J Bcl-2 expression in breast cancer: a comparative study at the mRNA and protein level Anticancer Res 2007 27 219 222 17352236

[B5] Ellis P Schnitt S Sastre-Garau X Bussolati G Tavassoli F Eusebi V Peterse J Mukai K Tabar L Jacquemier J Cornelisse C Sasco A Kaaks R Pisani P Goldgar D Devilee P Cleton-Jansen M Borresen-Dale A van't Veer L Sapino A Tavassoli FA & Devilee P WHO Classification of Tumours. Pathology and Genetics of Tumours of the Breast and Female Genital Organs 2003 Lyon 9 59

[B6] Perou C Sørlie T Elsen M van de Rijn M Jeffrey S Rees C Pollack J Ross D Johnsen H Akslen L Fluge Ø Pergamenschlkov A Williams C Zhu S Lønning P Børresen-Dale A-L Brown P Botstein D Molecular portraits of human breast tumours Nature 2000 406 747 752 10.1038/35021093 10963602

[B7] Sørlie T Perou C Tibshirani R Aas T Geisler S Johnsen H Hastie T Eisen M van de Rijn M Jeffrey S Thorsen T Quist H Matese J Brown P Botstein D Lønning P Børresen-Dale A-L Gene expression patterns of breast carcinomas distuinguish tumor subclasses with clinical implications Proc Natl Acad Sci 2001 98 10869 10874 10.1073/pnas.191367098 11553815 PMC58566

[B8] Foulkes W Smith I Reis-Filho J Triple-Negative Breast Cancer N Engl J Med 2010 363 1938 1948 10.1056/NEJMra1001389 21067385

[B9] Hanahan D Weinberg RA The hallmarks of cancer Cell 2000 100 57 70 10.1016/S0092-8674(00)81683-9 10647931

[B10] García M González-Reyes S González L Junquera S Berdize N Del Casar J Medina M Vizoso F Comparative study of the expression of metalloproteases and their inhibitors in different localizations within primary tumours and in metastatic lymph nodes of breast cancer Int J Exp Path 2010 91 324 334 10.1111/j.1365-2613.2010.00709.x PMC2962891 20412339

[B11] Nagase H Visse R Murphy G Structure and function of matrix metalloproteinases and TIMPs Cardiovascular Research 2006 69 562 573 10.1016/j.cardiores.2005.12.002 16405877

[B12] Hesek D Toth M Meroueh S Brown S Zhao H Sakr W Fridman R Mobashery S Design and Characterization of a Metalloproteinase Inhibitor-Tethered Resin for the Detection of Active MMPs in Biological Samples Chemistry & Biology 2006 13 379 386 10.1016/j.chembiol.2006.01.012 16632250

[B13] Deryugina E Quigley J Matrix metalloproteinases and tumor metastasis Cancer Metastasis Rev 2006 25 9 34 10.1007/s10555-006-7886-9 16680569

[B14] Del Casar J González-Reyes S González L González J Junquera S Bongera M García M Andicoechea A Serra C Vizoso F Expression of metalloproteases and their inhibitors in different histological types of breast cancer J Cancer Res Clin Oncol 2010 136 811 819 10.1007/s00432-009-0721-2 19916023 PMC11828129

[B15] Roy R Yang J Moses M Matrix Metalloproteinases As Novel Biomarkers and Potential Therapeutic Targets in Human Cancer J Clin Oncol 2009 27 5287 5297 10.1200/JCO.2009.23.5556 19738110 PMC2773480

[B16] Zhou P Du LF Lv GQ Yu XM Gu YL Li JP Zhang C Current evidence on the relationship between four polymorphisms in the matrix metalloproteinases (MMP) gene and breast cancer risk: a meta-analysis Breast Cancer Res Treat 2011 127 813 818 10.1007/s10549-010-1294-0 21161369

[B17] Vihinen P Kähäri V-M Matrix metalloproteinases in cancer: prognostic markers and therapeutic targets Int J Cancer 2002 99 157 166 10.1002/ijc.10329 11979428

[B18] Egeblad M Werb Z New functions for the matrix metalloproteinases in cancer progression Nat Rev Cancer 2002 2 161 174 10.1038/nrc745 11990853

[B19] Brinckerhoff C Rutter J Benbow U Interstitial Collagenases as Markers of Tumor Progression Clin Cancer Res 2000 6 4823 4830 11156241

[B20] McGowan PM Duffy MJ Matrix metalloproteinase expression and outcome in patients with breast cancer: analysis of a published database Ann Oncol 2008 19 1566 1572 10.1093/annonc/mdn180 18503039

[B21] Minn A Gupta G Siegel P Bos P Shu W Giri D Viale A Olshen A Gerald W Massague J Genes that mediate breast cancer metastasis to lung Nature 2005 436 518 524 10.1038/nature03799 16049480 PMC1283098

[B22] Eck S Hoopes P Petrella B Coon C Brinckerhoff C Matrix metalloproteinase-1 promotes breast cancer angiogenesis and osteolysis in a novel in vivo model Breast Cancer Res Treat 2009 116 79 90 10.1007/s10549-008-0085-3 18597171 PMC3772530

[B23] Vizoso FJ Gonzáles LO Corte MD Rodriguez JC Vazquez J Lamelas ML Junquera S Merino AM Garcia-Muniz JL Study of matrix metalloproteinases and their inhibitors in breast cancer Br J Cancer 2007 96 903 911 10.1038/sj.bjc.6603666 17342087 PMC2360108

[B24] Elston CW Ellis IO Pathological prognostic factors in breast cancer. I. The value of histological grade in breast cancer: experience from a large study with long-term follow up Histopathology 1991 19 403 410 10.1111/j.1365-2559.1991.tb00229.x 1757079

[B25] Boström P Söderström M Palokangas T Vahlberg T Collan Y Carpen O Hirsimäki P Analysis of cyclins A, B1, D1 and E in breast cancer in relation to tumour grade and other prognostic factors BMC Res Notes 2009 2 140 10.1186/1756-0500-2-140 19615042 PMC2716358

[B26] Köhrmann A Kammerer U Kapp M Dietl J Anacker J Expression of matrix metalloproteinases (MMPs) in primary human breast cancer and breast cancer cell lines: New findings and review of the literature BMC Cancer 2009 9 188 10.1186/1471-2407-9-188 19531263 PMC2706257

[B27] Heppner K Matrisian L Jensen R Rodgers W Expression of Most Matrix Metalloproteinase Family Members in Breast Cancer Represents a Tumor-Induced Host Response Am J Pathol 1996 149 273 282 8686751 PMC1865221

[B28] Baker E Stephenson T Reed M Brown N Expression of proteinases and inhibitors in human breast cancer progression and survival J Clin Pathol: Mol Pathol 2002 55 300 304 10.1136/mp.55.5.300 PMC1187260 12354933

[B29] Nakopoulou L Giannopoulou I Gakiopoulou H Liapis H Tzonou A Davaris P Matrix metalloproteinase-1 and -3 in breast cancer: correlation with progesterone receptors and other clinicopathologic features Hum Pathol 1999 30 436 442 10.1016/S0046-8177(99)90120-X 10208466

[B30] Duffy M Maguire T Hill A McDermott E O'Higgins N Metalloproteinases: role in breast carcinogenesis, invasion and metastasis Breast Cancer Res 2000 2 252 257 10.1186/bcr65 11250717 PMC138784

[B31] Przybylowska K Kluczna A Zadrozny M Krawczyk T Kulig A Rykala J Kolacinska A Morawiec Z Drzewoski J Blasiak J Polymorphismus of the promoter regions of matrix metalloproteinases genes MMP-1 and MMP-9 in breast cancer Breast Cacer Res Treat 2006 95 65 72 10.1007/s10549-005-9042-6 16267613

[B32] Coussens L Fingleton B Matrisian L Matrix Metalloproteinase Inhibitors and Cancer: Trials and Tribulations Science 2002 295 2387 2392 10.1126/science.1067100 11923519

[B33] González L Corte M Vázquez J Junquera S Sanchez R Alvarez A Rodriguez J Lamelas M Vizoso F Androgen receptor expression in breast cancer: Relationship with clinicopathological characteristics of the tumors, prognosis, and expression of metalloproteases and their inhibitors BMC Cancer 2008 8 149 10.1186/1471-2407-8-149 18507821 PMC2416360

[B34] Soerjomataram I Louwman M Ribot J Roukema J Coebergh J An overview of prognostic factors for long-term survivors of breast cancer Breast Cancer Res Treat 2008 107 309 330 10.1007/s10549-007-9556-1 17377838 PMC2217620

[B35] Cheng S Tada M Hida Y Asano T Kuramae T Takemoto N Hamada J-I Miyamoto M Hirano S Kondo S Moriuchi T High MMP-1 mRNA Expression is a Risk Factor for Disease-Free and Overall Survivals in Patients with Invasive Breast Carcinoma J Surgical Res 2008 146 104 109 10.1016/j.jss.2007.05.032 17663001

[B36] González L Corte M Junquera S González-Fernández R del Casar J García C Andicoechea A Vázquez J Pérez-Fernández R Vizoso F Expression and prognostic significance of metalloproteases and their inhibitors in luminal A and basal-like phenotypes of breast carcinoma Human Pathol 2009 40 1224 1233 10.1016/j.humpath.2008.12.022 19439346

